# The Apoptotic Properties of Leaf Extracts of *Simarouba glauca* against Human Leukemic Cancer Cells

**DOI:** 10.31557/APJCP.2021.22.4.1305

**Published:** 2021-04

**Authors:** Biba Vikas, Sujathan Kunjiraman, Suja Somasekharan Nair Rajam, Sukumaran Anil

**Affiliations:** 1 *Jawaharlal Nehru Tropical Botanic Garden and Research, Trivandrum, Kerala, India. *; 2 *Regional Cancer Centre, Trivandrum, Kerala, India. *; 3 *Department of Dentistry, Oral Health Institute, Hamad Medical corporation, Doha, Qatar.*; 4 *College of Dental Medicine, Qatar University, Doha, Qatar. *

**Keywords:** Simarouba glauca, cytotoxic, apoptotic, anticancer activity, antiproliferative, leukemia

## Abstract

**Background and objective:**

Simarouba glauca is a plant belonging to the family of Simaroubaceae. It is a potent source of secondary metabolites. The aim of this study was to evaluate the apoptotic properties of leaf extracts of Simarouba glauca against human leukemic cancer cells.

**Materials and Methods:**

Cytotoxicity of Simarouba glauca was assessed in the leaf extract of petroleum ether against leukemic cells by MTT assay. To detect the apoptotic features, fluorescence microscopy analysis was done with dual acridine orange/ethidium bromide fluorescent staining and Hoechst staining. To determine the externalization of phosphatidylserine, annexin v staining was done. Mitochondrial or death receptor activation was confirmed by caspase 3 analysis by flow cytometry.

**Results:**

This study revealed that Simarouba glauca was able to treat leukemia. Among the four extracts, petroleum ether extract showed a higher order of in vitro anticancer activity. The petroleum ether extract strongly inhibited the proliferation of K562 cell lines with IC_50_ values of 186 µg/ml. Dual acridine orange/ethidium bromide fluorescent staining and Hoechst staining revealed the characteristic features of apoptosis. Annexin V confirmed early and late stage apoptosis. Caspase-3 analysis revealed that cell death was due to mitochondrial or death receptor activation in mitochondrial pathway.

**Conclusion:**

These findings suggested that Simarouba glauca leaf extracts inhibited leukemic cells in a time- and dose-dependent manner either through mitochondrial or death receptor activation. The leaf extracts of Simarouba glauca was found to be nontoxic to lymphocytes. It can be concluded that Simarouba glauca is an important source of phytochemicals posing efficacy against leukemic cancer cells.

## Introduction

Cancer is a dreadful disease and a major public health problem in the world. Plant-derived drugs are used as one of the treatment for cancers. Plants have been used for medicinal purposes from the ancient period. The use of the medicinal plants in cancer prevention and management is common in all over world transmitted from generation to generation. Traditional medicines are widely practiced. Being cost effective and inducing lesser side effects are the benefits that led to use of plant materials as a source of medicines for various human ailments. Simarouba glauca is an evergreen edible oil tree belonging to family Simaroubaceae. This family includes 32 genera and more than 170 species of trees and bushes of pantropical distribution (Alves et al., 2014). Its common names are Lakshmi Taru and paradise-tree. A molecular phylogeny of the family was reported in 2007, data revealed with the relationships within the family (Clayton et al., 2007). 

Simaroubaceae family possess wide variety of chemicals and can be characterized as potential bioactive molecules with remarkable research potential. To substantiate the potentiality and reported in 1961. the first quassinoide structure was elucidated . More than 200 currently known chemicals are isolated and identified from Simaroubaceae family (Balu et al., 2020; Osagie-Eweka et al., 2021). Most of these secondary metabolites showed potential biological activities in bioassay systems posing structures which could be considered as effective therapeutic and bioactive agents (Vikas et al., 2017).

The cultivation of Simarouba Glauca is in semiarid dry and saline land areas of Indian states, such as Gujarat, Tamilnadu, Maharashtra, Karnataka, and Andhra Pradesh. Simarouba glauca tree usually grows in marginal wastelands, drylands, and degraded soil. This is an evergreen tree attaining a height 12-15 m and posing large circular crown. Simarouba glauca is grown under storey shade tolerant tree growing under the canopy of large fruit trees where birds perch and deposit the seeds near subtropical moist forest plants. This tree is a medium sized evergreen tree that begins to bear fruit when it is 6-8 years old and continues until 4-5 years later. Flowering takes place from December to February. This tree is polygamodioecious, and most of the female ones are good bearers. Bitter content substances in this tree has pharmaceutical properties (Fernando and Quinn, 1992; Muhammad et al., 2004).

The principal geographical distribution of this tree is at tropical America, extending to the west Africa, Madagascar, Asia (Malaysia), and some Pacific regions (Simão et al., 1991; Saraiva et al., 2002). This family is represented by the genera Quassia and Picrolemma in Brazil. Castela and Picrasma in Amazon, to the South and Simaba, Simarouba and Picrolema, which are present throughout the country (Arriaga et al., 2002; Almeida et al., 2007). Simarouba glauca is native to Southern Florida, the West Indies, and Brazil (Cronquist, 1944). It grows under tropical conditions in Central America spreading from Mexico to Panama Southern Florida and Caribbean Islands. Simarouba glauca was introduced in Kenya and Burundi in Africa in 1957 (Joshi and Joshi, 2002). 

The cultivation of Simarouba glauca extended to semiarid dry and saline land areas of other Indian states like Gujarat, Tamilnadu, Maharashtra, Karnataka and Andhra Pradesh. Simarouba glauca tree has an ability to grow well even in marginal wastelands and dry land with degraded soil (Govindaraju et al., 2009). This study highlighted the significance of anti-cancer and apoptotic effect of petroleum ether extracts (LPEs) of Simarouba glauca on leukemic cancer cells.

## Materials and Methods

**Figure 1:**
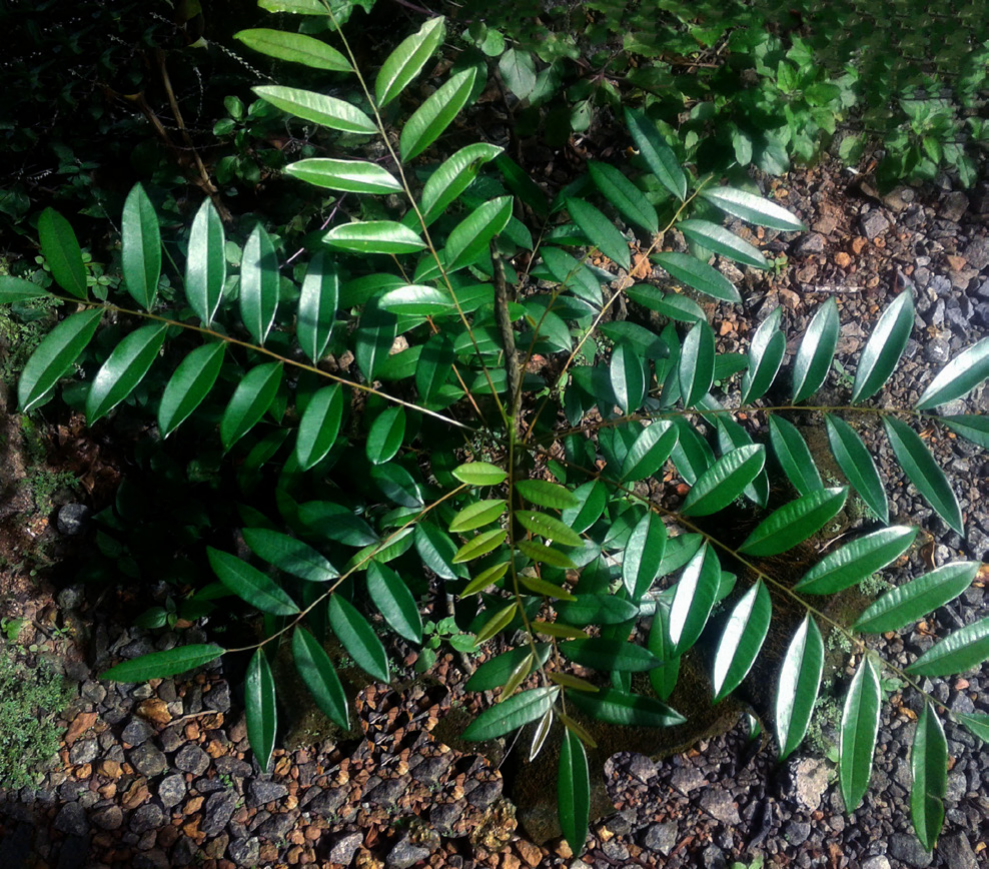
*Simarouba glauca* Plant

Preparation of leaf extract: The leaf extracts of Simarouba glauca Voucher specimen 95212 was authenticated by the Taxonomist and deposited at JNTBGRI Herbarium. (Figure 1) were collected and shade dried and pulverized seeds were used for soxhlet extraction in a soxhlet apparatus using Petroleum ether (LPE), chloroform (LCH), ethyl acetate (LEA) and methanol (LME) as the solvents and concentrated by using rotatory evaporator. The leaf extracts of Simarouba glauca were dissolved in dimethyl sulfoxide (DMSO) at a concentration of 10μg/ml and stored at 20ºC. Appropriate dilutions of extracts were prepared in culture medium immediately before the experiments.

Cell lines and culture conditions: Leukemic cancer cells (K-562 ) were obtained from National Centre for Cell Sciences (NCCS) Pune and grown in DMEM media supplemented with 10 % FBS, HEPES buffer, penicillin (100 units/ml), and streptomycin (100 μg/ ml). Cells were maintained at 37^o^C in a humidified atmosphere with 5% CO_2_ .

Qualitative phytochemical analysis: Various qualitative chemical tests were performed for establishing profile of four extracts and determining their chemical composition. The Simarouba glauca leaf (L) extract, petroleum ether extract (LPE), Chloroform extracts (LCH), Ethyl Acetate extract (LEA), and Methanol extract (LME) were analyzed for the presence of various phytoconstituents by following standard phytochemical tests.

Steroids/ terpenoids– Liebermann-Burchard reaction (LB): Freshly prepared reagent (5 ml acetic anhydride and 5 ml Sulfuric acid) was added to the various extracts. Appearance of a pink color showed the presence of terpenoid ; whereas, a green colour indicated the presence of steroids (Richardson and Harborne, 1985).

Shinoda’s test for flavonoids:About1 mg of each extract was dissolved in 5 ml of methanol. Then, magnesium powder and 5M of hydrochloric acid were added . The appearance of pink color indicated the presence of flavonoids. Flavonoids are a group of about 4000 naturally occurring poly phenolic compounds, found universally in foods of plant origin (Harborne, 1986)). 

Detection of alkaloids: Solvent-free plant extract was stirred with 0.1ml of dilute hydrochloric acid and then was filtered. The filtrate was tested carefully with various alkaloid reagents as follows (Kintzios, 2007).

Wagner’s test: To 1ml of filtrate, 0.5 ml of Wagner’s reagent was added to the test tubes. A reddish- Brown precipitate confirmed the test as positive . 

Wagner’s reagent: Iodine (1.27g) and potassium iodide (2g) were dissolved in 5ml of distilled water and the solution was made up to 100 ml with distilled water was added to 1ml of the extracts dissolved in methanol. Alkaloids gave brown flocculent precipitate. 

Detection of cytotoxicity/ antiproliferative activity by cell viability assay (MTT assay). The sample was taken at different concentrations and tested for in vitro cytotoxicity on the leukemic cancer cell lines by MTT assay (Scudiero et al., 1988)). 5 x 103 cells were plated in 100 µl of the medium in 96-well plates in the presence or absence of various concentrations of the extracts in different schedules. At the end of the incubation, 25 µl of MTT solution was added to each well. After 2-hour incubation, at 370 C, 100 µl of the extraction buffer was added in 50% dimethylformamide. After 4-hour incubation, the optical density at 570 nm was determined using an ELISA plate reader with the extraction buffer as blank (Anto et al., 2000). 

Detection of apoptosis: Most of the anti-proliferative agents induce programmed cell death (apoptosis) in transformed cells, and this is one of the best ways to eliminate the malignant cells. Hence, the principle components were tested for the induction of apoptosis in leukemic cells. 

Determining morphological features of apoptosis by Acridine Orange – Ehidium Bromide dual staining: Staining was done to analyze the morphological features of apoptosis like membrane blebbing, chromatin condensation, and formation of apoptotic bodies etc. (Kerr et al., 1994). The apoptosis can be detected by using acridine orange ethidium bromide dual staining. Cells were cultured in 24 multiwell titre plates. 1x 106 cells were incubated in 10 % FBS containing DMEM media with various concentrations of the extract and incubated in a CO_2_ incubator at 37oC (Kirsch-Volders et al., 1997). Apoptosis assessment can be done 24 hours after exposure of cells to the active extracts. Acridine orange/ethidium bromide double staining was used to identify apoptosis-associated changes in this study. DNA dying made the visualization of the condensed chromatin of apoptotic cells possible. The media was removed and then cells were pelleted gently. Next, 1 µl of acridine orange and 1 µl of ethidium bromide in 1ml PBS were added to the cells and immediately cells were washed with PBS. Cells were then re-suspended in 10 µl of 10 percentage glycerol in PBS and the slides were viewed under a fluorescent microscope. The number of cells showing morphological features of apoptosis were counted as a function of the total cells present in the field.

Hoechst 33342 staining: The LPE treated cells (10 μg/ml and 15 μg/ml) for 24 hours were washed twice with PBS and stained with Hoechst 33342 at room temperature. The Hoechst stained nuclei were visualized by fluorescence microscope with emission 350- 460 nm.

Detection of annexin status by flow cytometry: Treated and untreated cells with extracts were harvested, and 2x105 cells were washed with PBS and re-suspended in binding buffer (i.e. 10mM HEPES, 150mM NaCl, 5mM KCl, 1mM MgCl_2_, 1.8mM CaCl_2_; Ph 7.4). Propidium Iodide (2μg/ ml final) and annexin V conjugated with fluorescein isothiocyante (FITC) were added to differentiate necrotic cells ( PI positive ) from apoptotic and live cells ( PI negative ). Cells were diluted into 1 ml PBS then pelleted and re-suspended in 1ml PBS. Cells were analyzed immediately by fluorescence activated cell sorting (FACS- Becton- Dickinson). Cells with FITC/ PI positive (necrotic) were excluded from the analysis.

Flow cytometry detection of caspase: Caspase analysis was done on cells treated with and without the active extract to detect apoptosis, mitochondrial, or death receptor pathways. The cells were cultured and then washed with PBS. Cells were diluted into 1 ml PBS, pelleted, and then re-suspended in 1ml PBS. Cells were analyzed immediately by fluorescence- activated cell sorting (FACS- Becton- Dickinson).

*Statistical analysis*

The results were represented as mean + SD. The data were analyzed by using excel and Easyplot.

## Results

Phytochemical analysis: The leaf extraction of Simarouba glauca using a selected panel of solvents gave four extracts, namely LPE, LCH, LEA, and LME . These four extracts underwent phytochemical evaluation. Phytochemical screening of the LPE, LCH, LEA, and LME extracts revealed that they contained bioactive chemical substances, such as flavonoids, alkaloids, and terpenoids but not saponins (Table 1).

**Figure 2:**
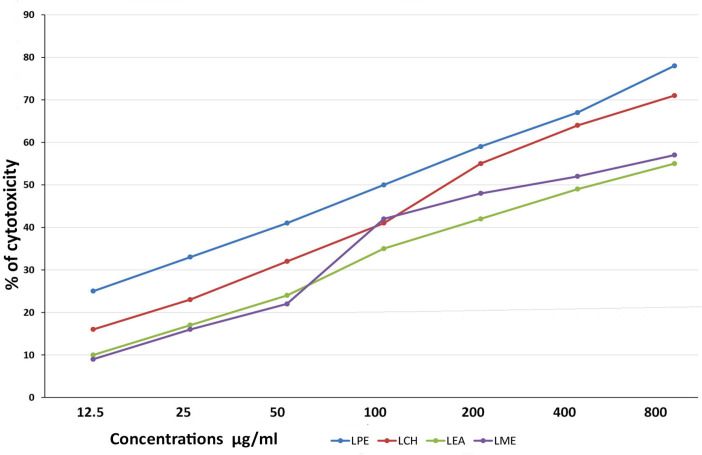
Screening of Simarouba Gluaca Leaves Extracts such as Petroleum ether, Chloroform, Ethyl Accetate and Methanol on K-562 Cells

**Figure 3:**
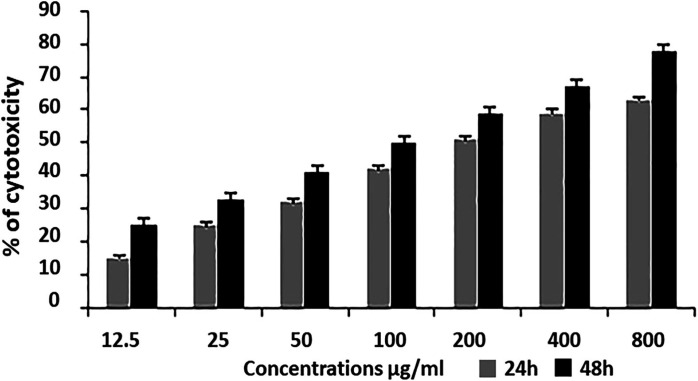
MTT Assay: Leaves of *Simarouba gluaca* Petroleum Ether Extract Induced Cell Death in Leukemic Cells

**Figure 4:**
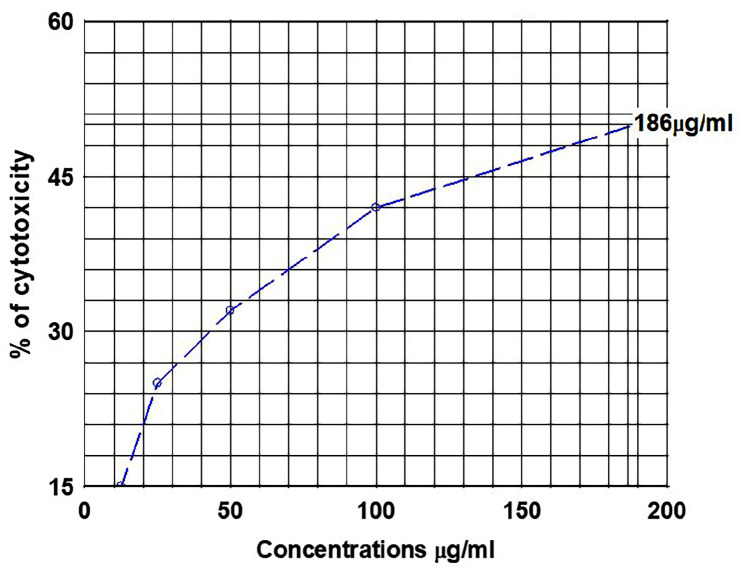
IC_50_ Value of LPE Induced in Leukemic Cells after 48 Hours

MTT assay for K562 cell line: When four extracts were evaluated for cytotoxicity against K-562 cell line, LPE showed maximum cytotoxicity of 78%, LCH 71%, LEA 55%, and LMet 57% at a concentration of 800µg/ml and within 48hrs (Figure 2). In nutshell, LPE and LCH showed maximum cytotoxicity. Hence, further investigation was conducted on LPE. Treatment with LPE and LCH at concentrations of 12.5 μg/ml to 800μg/ml and within 24 to 48 hours induced inhibition of cell proliferation to 63± 1.78%±2, respectively, in K562 cells (Figure 3). The IC50 values of K562 were found to be 186 µg/ml (Figure 4). 

**Figure 5:**
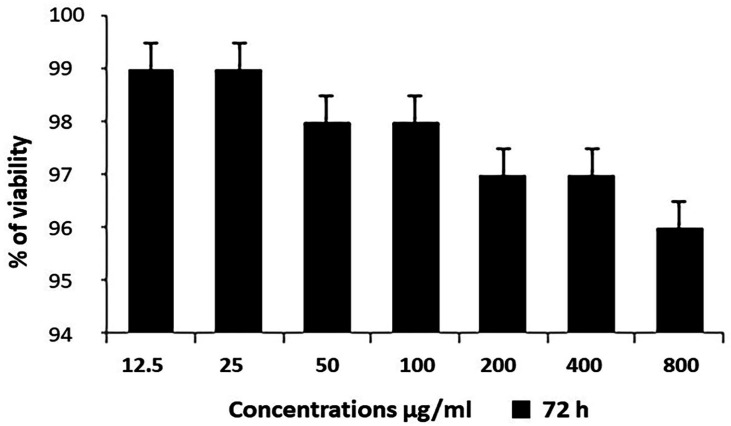
Lymphocyte Viability Assay: Normal lymphocytes treated with various concentrations of simarouba gluca petroleum ether extracts

Lymphocyte viability assay: To ascertain whether the LPE specifically inhibit the proliferation of dividing cells, its effect was assessed against human lymphocytes culture. The viability of cells after 72 hours was 99±0.5% - 96±0.5% at concentrations ranging from 12.5 to 200µg/ml (Figure 5). No significant toxicity was found after the treatment.

**Figure 6:**
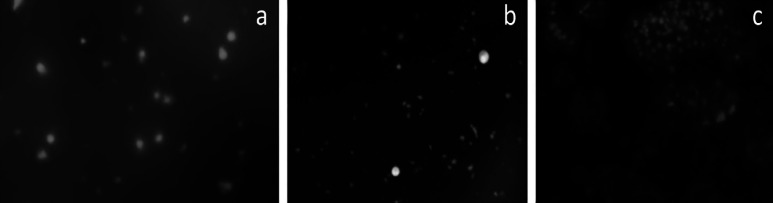
Acridine Orange/Ethidium Bromide Staining: a, Untreated K-562 control cell; b, K-562 cells treated with LPE 10µg/ml; c, K-562 cells treated with LPE 15µg/ml

Morphological features of dual acridine orange/ethidium bromide fluorescent staining: The results showed a dose-dependent increase in the percentage of apoptotic cells following treating with LPE. Dual acridine orange/ethidium bromide fluorescent staining revealed the characteristics of apoptosis, namely membrane blebbing, condensation of chromatin, and nuclear fragmentation. K562 cells exposed to LPE 10μg/ml and LPE 15μg/ml within 24 hours showed apoptotic features like condensed chromatin and fragmented nuclei (Figure 6). Control cells appeared with the nuclear region uniformly green . 

**Figure 7:**
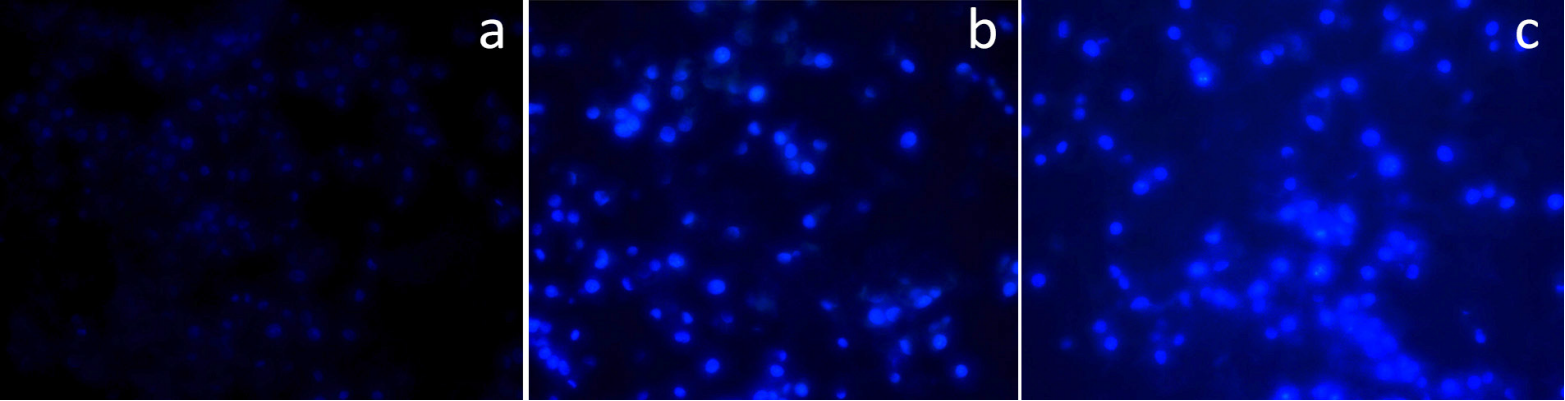
Hoechst Staining: a, Untreated K-562 control cells; b, K-562 cells treated with LPE 10µg/ml; c, K-562 cells treated with LPE 15µg/ml

Investigating cell death by Hoechst staining: Hoechst dye was able to diffuse through intact membranes of K562 cells and stain their DNA. Figure7 shows the results of Hoechst staining for the 10μg/ml and 15μg/ml LPE treated K562 cells at different concentrations and within 24 hours. 

**Figure 8.**
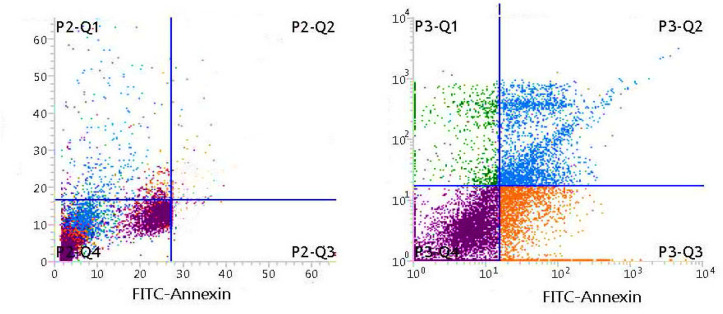
Annexin V Staining by Flowcytometry: a, Untreated K-562 control cells; b, K- 562 cells treated with LPE 10µg/ml

Apoptotic effects by the translocation of phosphatidylserine : 10μg/ml and 15μg/ml LPE treated cells showed increased annexin V expression. Number of annexin V positive cells treated with 10μg/ml LPE for K-562 was 65% (Figure 8). 

**Figure 9:**
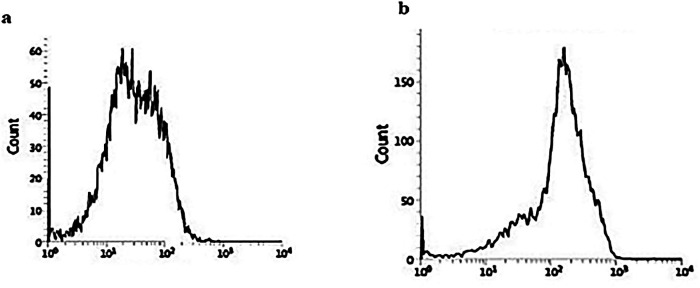
Caspase Assay by Flow Cytometry of LPE 10µg/ml Treated K-562 Cells

Elevated expression of caspase-3: 10μg/ml and 15μg/ml LPE induced caspase-3 activation. Results from the cytotoxicity studies, apoptosis studies by Annexin V binding once again indicated the apoptotic induction of 10μg/ml in K-562 is 67 % LPE (Figure 9).

**Table 1:** Phytochemical Screening of Different Extracts of Leaves of *S. Glauca*

Constituents	ASPE	ASCH	ASEA	ASME
Flavanoids	+	+	+	_
Alkaloids	-	+	+	+
Terpenoids	-	-	+	+
Saponins	_	_	_	_

## Discussion

Medicinal plants are important resources of phytochemicals and herbal drugs. Pharmaceutical companies have screened more than 25,000 plants for developing anti-cancer drugs. Herbal system of medicine has been applied for thousands of years (Sakarkar and Deshmukh, 2011). Derivatives of natural products represent approximately 60% of all chemotherapeutic agents approved by the Food and Drug Administration (FDA); for example, vincristine, vinblastine, and Taxol (Newman, 2014; Katz and Baltz, 2016; Newman and Cragg, 2016; Florian et al., 2020). The process of programmed cell death, apoptosis, is characterized by morphological features and biochemical mechanisms. Apoptosis is assumed as an important component of various processes, including normal cell turnover, proper development and functioning of the immune system, hormone-dependent atrophy, embryonic development, and chemical-induced cell death (Elmore, 2007). Dead cells generated due to apoptosis are quickly engulfed by macrophages for degradation. Caspases are a large family of cysteine proteases that act in cascades. A cascade that leads to caspase 3 activation mediates apoptosis and is responsible for killing cells, recruiting macrophages, and presenting an “eat me” signal(s) (Nagata, 2018). In this study, we evaluated the anti-cancer and apoptotic properties of Simarouba glauca belonging to the Simaroubaceae family. Many compounds isolated from different members of this family have been previously reported to have anticancer properties (Yeo et al., 2014; Jose et al., 2018). In this study, four different extracts were obtained from the leaf of Simarouba glauca . The extracts were screened for cytotoxicity using MTT assay. Maximum cytotoxicity was observed at the highest concentration of 800µg/ml in SG extract on leukemic cells. The findings of this study indicated that MTT assay offered the convenience of providing drug sensitivity information.

Given that appropriate cytotoxicity was proved for cancer cell line treated with LPE of SG, further investigation was carried out to assess the effect on normal cells. Following lymphocyte viability assay, no significant toxicity was observed for normal cells up to the concentration of 800µg/ml. Accordingly, it was concluded that this extract (SGPE) could kill cancer cells by sparing normal cells.

Apoptosis is characterized by a number of characteristic morphological changes in the structure of the cell, along with a number of enzyme-dependent biochemical processes (Pulok et al., 2020). 

Different fluorescence staining procedures were conducted on the leukemic cells treated with different concentrations of SGPE to determine the cell death by apoptosis and discover apoptotic features. On acridine/ethidium bromide staining, the acridine orange entered the live cell and intercalated the DNA at the G-C base pairs, while the ethidium bromide entered the dead cell and intercalated the DNA at the A-T base pairs. The viable cells fluorescence green and the dead cells fluorescence orange. Apoptotic features, such as chromatin condensation, membrane disruption, and membrane blebbing, were observed.

Hoechst stain confirmed the death of cells by apoptosis through showing bright blue fluorescence as a result of Hoechst stain binding to the A-T base pairs. Apoptotic features like nuclear condensation and DNA fragmentation were observed. Being hydrophilic in nature, Hoechst stain enters the cell only when there are lesions on the cell surface and hence is an indicator of membrane integrity suggesting that the cells that gave bright blue fluorescence on staining have started undergoing apoptosis or is at the end of the staining procedures does confirm the death of the cells by apoptosis. 

We utilized double labelling techniques to understand the nature of cell death. In addition, we used fluorescent labelled Annexin V/ Propidium Iodide to differentiate between apoptotic and necrotic cells. In the early stages of apoptosis, the cell membrane was intact, the cells were impermeable to propidium iodide but permeable to Annexin. Afterwards in the later stages of apoptosis, it was permeable to both dyes. On the contrary, the necrotic cells were permeable only to PI and not to Annexin as there was no phosphatidyl serine translocated to the extracellular leaflet of the membrane to which Annexin binds. Flow cytometric data revealed that both dyes entered in SGPE treated cells in a dose and time dependent manner, inferring the induction of late stages of apoptosis by SGPE on the cancer cells. The two major mechanisms of cell death through apoptosis are intrinsic and extrinsic pathways. These pathways lead to a cascade of events and consequently the activation of an effector enzyme, caspase-3. Caspase-3 is a cysteine protease with aspartic specificity and the most well-characterized effector caspase that executes apoptosis. Conversely, its over-activation can cause excessive programmed cell death. The active caspase-3, labelled with fluorochrome-conjugated anti-activated caspase-3 antibodies and analyzed by flow cytometry SGPE 12.5µg/ml treated leukemic cells , showed elevated caspase-3 expression. Accordingly, it can be concluded that apoptosis induction was activated through either mitochondrial or death receptor pathway. Altogether, LPE of Simarouba glauca showed cytotoxicity with no significant toxicity towards normal cells and was able to induce apoptosis in leukemic cells.

In the present study, Simarouba glauca was selected from the Simaroubaceae family. Four different extracts were obtained using four different solvents. Their cytotoxicity against leukemic cell line was determined using MTT assay. The LPE of Simarouba glauca showed maximum cytotoxicity and hence it underwent further investigation. The cytotoxicity of SGPE against leukemic cell line was determined using MTT assay and maximum percentage of cytotoxicity was detected. Lymphocyte viability assay was conducted using SGPE and no significant death in the normal cells was found but it showed more specificity towards cancer cells. For further fluorescence staining like acridine orange-ethidium bromide and hoechst staining of SGPE treated against leukemic cells showed apoptotic cell death. Further, the early and late stages of apoptosis, death of cells was evaluated and confirmed by Annexin-v staining. Caspase-3 expression revealed that the pathway of SGPE was blocked through either mitochondrial or death receptor pathway. In conclusion, the study revealed that petroleum ether leaf extract of the Simarouba glauca induced cell death through apoptosis. It seems that Simaroubaceae family contains compounds that can have effective anticancer properties.

## Author Contribution Statement

BV and SA-Conceived and designed the analysis; BV-Collected the data; BV, SR, KS-Contributed towards analysis and interpretation of data; BV and SA -Performed the analysis; BV-Wrote the paper, SA and KS edited the paper. All authors read and approved the manuscript.
